# Next-Generation Spectacle Lenses for Myopia Control: Optical Designs, Mechanisms, and Clinical Efficacy

**DOI:** 10.3390/jcm14217872

**Published:** 2025-11-06

**Authors:** Neeraj K. Singh, Pablo De Gracia

**Affiliations:** 1 Kentucky College of Optometry, University of Pikeville, Pikeville, KY 41501, USA; 2 School of Optometry, University of Detroit Mercy, Detroit, MI 48377, USA; degracpa@udmercy.edu

**Keywords:** myopia, spectacle lenses, myopic defocus, optical interventions

## Abstract

Myopia prevalence has risen dramatically worldwide, underscoring the critical need for effective interventions to slow its progression. Recent advancements in spectacle lens technology offer promising solutions, demonstrating significant efficacy in controlling myopia. This review critically examines next-generation spectacle lenses for myopia management, emphasizing their optical principles, mechanisms of action, clinical effectiveness, visual performance, compliance, and safety. Spectacle lenses incorporating technologies such as Defocus Incorporated Multiple Segments (DIMS), Highly Aspherical Lenslet Target (HALT), Diffusion Optics Technology (DOT), and Cylindrical Annular Refractive Element (CARE) lenses show a 40–60% reduction in refractive progression and axial elongation compared to traditional single-vision lenses. These lenses utilize optical strategies like simultaneous myopic defocus, peripheral contrast modulation, and controlled aberrations without compromising visual acuity, contrast sensitivity, accommodation, or binocular vision. High wearer compliance is attributed to excellent visual comfort, minimal adaptation issues, and favorable cosmetic appearance. Long-term studies further confirm sustained efficacy and safety profile. Ongoing research aimed at direct comparative trials, extended follow-up, and individualized lens designs will further define the role of these interventions. Collectively, the evidence positions next-generation spectacle lenses as a promising, evidence-based approach that may become an important component of global myopia management.

## 1. Introduction

Myopia has reached epidemic proportions worldwide, with projections that roughly half of the global population may be myopic by 2050 [1]. High myopia (≥−5.00 D) is raising the long-term risks of pathologic ocular complications such as myopic macular degeneration, glaucoma, and retinal detachment [2]. In this context, slowing myopia progression during childhood has become a priority to reduce future vision-threatening sequelae. Evidence-based myopia control interventions now include pharmacologic therapy (e.g., low-dose atropine [3]) and optical treatments such as orthokeratology [4] and dual-focus contact lenses [5]. Until recently, spectacle lenses offered limited myopia control efficacy—for example, progressive addition lenses (PALs) produced only small, clinically negligible slowing of myopia progression [6,7]. Traditional bifocal spectacles (e.g., executive bifocals) showed moderate effects (~30–40% reduction in progression in some studies) [8,9], but their mechanism was unclear and the cosmetic and binocular vision implications of lined bifocals limited widespread use in children. Early attempts at peripheral defocus spectacle designs did not yield significant myopia control benefit in clinical trials [10].

Unlike these earlier approaches, which relied on near additions or crude peripheral defocus, next-generation designs employ structured optical elements—such as lenslets, diffusers, or annular optics—that deliver a consistent myopic defocus or contrast signal while preserving central clarity. These innovations have enabled the development of new-generation spectacle lenses that demonstrate clinically meaningful efficacy, comparable to other interventions like orthokeratology and multifocal contact lenses. These novel designs incorporate specialized optical elements to impose myopic defocus or alter retinal image quality across the visual field while maintaining clear central vision. For example, the Defocus Incorporated Multiple Segments (DIMS) [11] lens and the Highly Aspherical Lenslet Target (HALT) [12] lens have each been shown in randomized trials to slow myopic eye growth by approximately 50% on average. Other approaches include diffusion optics technology (DOT) [13], which uses micro-diffusers to modulate contrast, and cylindrical annular refractive element (CARE) [14] lenses, which use concentric microstructure rings to create defocus zones.

This review provides an in-depth examination of spectacle lenses for myopia control, including their optical design principles and proposed mechanisms of action, and evaluates clinical efficacy and safety outcomes from major studies around the world. We compare leading designs (e.g., Hoya MiYOSMART with DIMS technology, Essilor Stellest with HALT technology) using peer-reviewed trial data and discuss visual performance, patient acceptance, and long-term results. We also summarize evidence from large-scale trials and long-term follow-ups to assess sustained efficacy across populations. The goal is to furnish clinicians and researchers with a comprehensive understanding of the state-of-the-art in myopia control spectacles, as a scientifically rigorous and globally relevant resource.

## 2. Optical Principles of Myopia Control Spectacle Lenses

**Figure 1:**
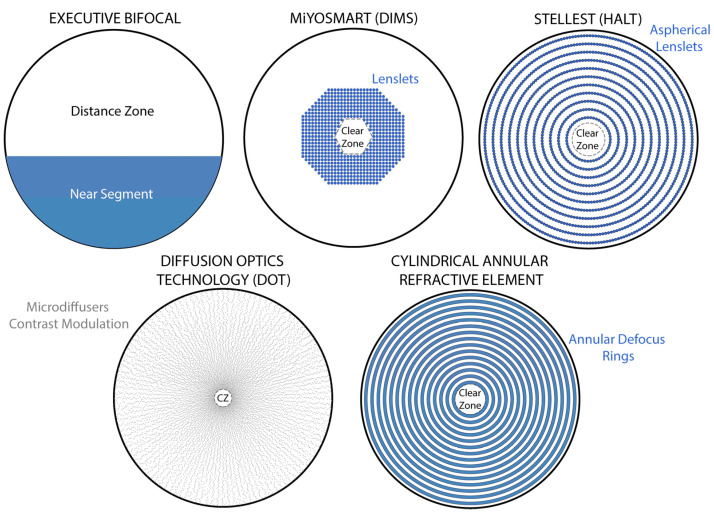
Illustrations of five different spectacle lens designs for myopia control. In the top row (from left to right), the Executive bifocal with a lined near segment is shown, which was historically employed with moderate success; alongside it, the Hoya MiYOSMART lens uses the DIMS (Defocus Incorporated Multiple Segment) design, embedding a honeycomb array of small plus-power lenslets in the mid-periphery; and the Essilor Stellest lens employs the HALT (Highly Aspherical Lenslet Target) design, with numerous contiguous aspherical micro-lenslets arranged in concentric rings. Both MiYOSMART and Stellest maintain a large central clear zone for distance vision. In the bottom row (from left to right), the SightGlass DOT (Diffusion Optics Technology) lens incorporates thousands of microscopic light-scattering elements distributed across the periphery to reduce retinal image contrast, based on the contrast modulation theory of myopia control, while leaving a small central zone clear; next to it, the ZEISS MyoCare lens applies the CARE (Cylindrical Annular Refractive Element) design, in which alternating concentric annular refractive rings (“donut-shaped” elements) provide myopic defocus interleaved with clear zones across the lens periphery, while maintaining an 8–10 mm central optic free for distance vision.

Modern myopia control spectacles share a common goal: to present the eye with simultaneous myopic defocus in addition to the normal corrected image, thereby signaling the retina to slow axial growth, a concept derived from the early studies on animal models [15,16,17,18]. Unlike a standard single-vision lens which brings all light to focus on the retina, these specialized lenses create a dual focal condition [19]. One focal plane (the clear central optical zone) corrects the refractive error on the fovea, while secondary focal points or defocused light are engineered to fall in front of the retina (myopic defocus) in the peripheral or parafoveal field [20]. This optical principle leverages animal model evidence that peripheral or simultaneous myopic defocus can act as a “stop signal” to ocular elongation [15,18]. The central challenge in the design of these lenses is achieving a therapeutically meaningful level of myopic defocus without compromising visual quality or comfort in real-world viewing conditions (Figure 1).

### 2.1. Defocus Incorporated Multiple Segment (DIMS) Technology

The DIMS design (commercialized as MiyoSmart^®^ (Hoya Lens, Tokyo, Japan)) incorporates small plus-powered lenslets (micro-segments) across the mid-periphery of an otherwise single-vision lens. Specifically, the MiYOSMART DIMS lens features a 9 mm central clear zone for distance vision, surrounded by a “honeycomb” matrix of approximately 400 tiny lenslets of +3.50 D add power, each ~1 mm in diameter [21]. These lenslets are discrete and cover the lens annular zone typically from about central radius outward, providing a pattern of simultaneous myopic defocus when the wearer views through the lens periphery. The power (+3.50 D) and placement of the segments are such that light passing through them focuses in front of the retina (approximately 0.7 mm anterior to the retinal plane) while the central line of sight remains clearly focused on the retina [21]. In effect, wherever the eye looks, it experiences a portion of the visual field in sharp focus and another portion in myopic defocus [19]. This simultaneous vision principle ensures that the treatment signal (myopic defocus) is present at all viewing distances without requiring the child to consciously look through different zones as in a bifocal. The DIMS lenslets act as “distance” segments (not near adds) and have been shown not to significantly alter vision function [22]. By distributing small defocus segments in a high-density array, the DIMS design aims to minimize perceptible image ghosting or distortions. Clinical studies have reported that children adapt well to DIMS lenses [23], with no significant complaints of blur or discomfort and visual acuity [24].

### 2.2. Highly Aspherical Lenslet Target (HALT) Technology

The HALT design (Stellest^®^ (Essilor, Charenton-le-Pont, France)) [25] pushes the concept of lenslets further by using aspherical micro-lenslets arranged in concentric rings. The Stellest lens has approximately 1021 lenslets divided across 11 rings around a 9 mm central clear zone [19]. Unlike the uniform +3.50 D power of DIMS, the HALT lenslets have varying power and asphericity by ring—each ring’s lenslets share the same design, but moving from the center outwards, the geometry is adjusted to create a gradient of defocus power. All lenslets in Stellest are contiguous within each ring, maximizing the area delivering the treatment signal. The innovative aspect of HALT is the creation of a “volume of myopic defocus” (VoMD) rather than a single add power plane [26]. The highly aspheric lenslets spread the defocused light over a range of dioptric powers in front of the retina—conceptually forming a three-dimensional shell of defocused light (for example, spanning ~0.7 mm in depth and 1.2 mm in front of the retina for the HALT design) [26]. This volume of defocus is hypothesized to provide a cue to slow eye growth, rather than a single focal plane [19]. In essence, the HALT lens simultaneously presents the eye with both clear imagery and a cloud of slightly blurred light in front of the retina [20]. The eye’s growth feedback mechanism is believed to respond predominantly to the myopic defocus component. Like DIMSs, Stellest lenses maintain good visual function [27].

### 2.3. Diffusion Optics Technology (DOT)

An alternative strategy to create a myopic defocus signal has been proposed through contrast modulation rather than dioptric defocus [28]. Diffusion Optics Technology (DOT), by SightGlass Vision, Dallas, TX, USA [29], embeds thousands of microscopic light-scattering elements (“diffuser dots”) into the lens material. The DOT lens does not have discrete plus-powered segments; instead, the micro-diffusers (each approximately 0.14 mm in size) are distributed across the lens periphery to scatter a small portion of light and thus reduce image contrast on the retina in a controlled manner [30]. The premise is rooted in the “contrast theory” of myopia: high image clarity and contrast at the retina (especially in indoor, close-up viewing) may encourage eye growth, whereas the natural outdoor visual environment—which features lower contrast, more depth of field, and a mix of focal distances—is protective against myopia [31]. By mimicking aspects of the outdoor visual experience (slightly degraded high-frequency contrast) even when a child is wearing glasses indoors, the DOT lens aims to slow myopic progression [28]. The DOT lenses have a smaller central clear aperture (~5 mm for distance gaze) to ensure some peripheral rays always encounter the diffusers. Two DOT lens configurations were explored: one with diffuser elements spaced relatively farther apart (DOT 0.2, causing a mild contrast reduction), and another with elements closer together (DOT 0.4, causing greater light scatter) [32]. The diffusive elements are engineered with two different refractive indices within the lens, scattering primarily certain wavelengths to selectively affect L- and M-cone photoreceptors responsible for high-detail vision [32]. By slightly raising the “background blur” across the retina, the DOT lens provides a uniform myopic blur signal without introducing dioptric power shifts. Clinical trials have shown that DOT lenses offer a visual experience comparable to standard single vision lenses, suggesting they are unlikely to negatively impact a child’s everyday activities [33].

### 2.4. Cylindrical Annular Refractive Element (CARE) Design

Another recent entrant is the CARE spectacle lens (MyoCare^®^, Zeiss Vision Care, Aalen, Germany) [34], which employs concentric refractive rings to create alternating focal zones. The CARE lens contains micro-structures known as Cylindrical Annular Refractive Elements that form a series of ring-shaped zones across the lens periphery [34]. These rings alternate with normal refractive zones in approximately a 50:50 ratio over the treatment area. In effect, the design creates concentric annular “donut-shaped” lenslets that provide +defocus power in the ring zones, interleaved with clear zones for vision [35]. The central optic is typically clear for distance vision (on the order of 8–10 mm diameter, as the exact size may vary with lens power and pupil considerations). The cylindrical annular elements differ from spherical lenslets by spreading the addition power in an annular fashion, which is theorized to preserve better optical quality along certain meridians while still introducing myopic defocus in the retinal periphery [35]. Essentially, the CARE approach is another way to provide simultaneous defocus: as the eye looks through the lens, portions of the pupil will sample light from the defocus rings (imposing myopic blur), while other portions sample the clear zones (providing the clear image) [35]. Early user reports indicate 98% compliance and overall satisfaction among children with CARE lenses [36] (Table 1).

## 3. Mechanisms of Myopia Control with Spectacle Lenses

### 3.1. Myopic Defocus as a Growth-Inhibiting Signal

The primary mechanism by which specialized lenses slow myopia progression is believed to be the introduction of myopic defocus [15]. In a normally corrected myopic eye viewing distant objects, the peripheral retina may actually be hyperopically defocused (light focusing behind the retina off-axis) even when central vision is clear [37]. This peripheral hyperopic defocus is thought to stimulate axial elongation as the eye “grows” to catch up to the peripheral image shell. Myopia control lenses counteract this by deliberately shifting part of the image in front of the retina (myopic defocus), which research in animal models and humans indicates sends a stop signal to eye growth [18]. Both DIMS and HALT lens designs explicitly create simultaneous defocus: the eye receives competing images (one in focus, one in front of the retina), and the retina may respond to myopic defocus as a signal to slow axial growth and achieve emmetropization [38]. The precise biological pathway is still under investigation, but evidence suggests that retinal neurons (potentially in the peripheral retina) detect the sign of defocus and modulate scleral growth rates accordingly. Imposing myopic defocus across a wide area of the retina (as performed by ring lenslets or scattered segments) may more effectively engage this anti-growth signal than, for example, a simple center-near add in a PAL (which primarily affects near gaze only). Importantly, studies have shown that the eye’s accommodative behavior is not adversely affected by these defocus designs—children do not adjust focus to “clear” the imposed blur, because the blur zones are simultaneous and peripheral (for example, dual-focus contact lens designs) [39]. Thus, the intended myopic blur signal remains consistently present to modulate eye growth even after sustained near viewing [40].

### 3.2. Peripheral Retinal Image Quality and Aberrations

Higher-order aberrations have been suggested to play a potential role in modulating peripheral image quality and signaling mechanisms involved in myopia control. In normal single-vision correction, while central vision is sharp, off-axis rays can produce peripheral hyperopic blur [16]. The lenslet designs of DIMS, HALT, and CARE effectively introduce controlled amount of plus power (myopic defocus) that alter the peripheral focus profile of the eye [41]. By doing so, these lenses may reduce the relative peripheral hyperopia and even induce slight myopic astigmatism or spherical aberration that the eye interprets as a cue to slow growth. It has also been hypothesized that higher-order aberrations induced by the micro-optic design, such as increased spherical aberration or coma, may contribute to retinal signaling mechanisms involved in myopia control [42]. The rationale is that a certain degree of retinal blur (beyond just defocus) might be “sensed” by retinal cells—for instance, degraded image quality on the peripheral retina might mimic the effects of extended depth of field experienced outdoors, which is linked to less myopia progression [43]. While myopic defocus is considered the dominant mechanism, optical quality changes such as reduced contrast, aberrations) may play a contributory role in modulating axial eye growth [44,45]. The DOT lens is an example of leveraging retinal image quality that operates by lowering retinal image contrast uniformly, which in theory could reduce the stimulus for axial elongation associated with chronically high contrast/foveal-centric tasks [30,33]. The successful clinical results of DOT lenses (slowing myopia with no dioptric add power at all) strongly support the concept that contrast and focus cues together influence eye growth [32]. In summary, myopia control spectacles work by altering the visual environment of the eye—through myopic defocus, through spatial contrast (diffusion), and through optical aberrations—to provide biological feedback that counters the signals driving excessive axial elongation.

### 3.3. Role of Accommodation and Binocularity

A secondary consideration is how these lenses interact with the accommodative and binocular vision system. Unlike PALs or bifocals, DIMS/HALT lenses do not provide a traditional near addition and it is unclear if and how the constant defocus zones affect accommodative behavior or convergence. Changes in binocular vision after 24 months have been reported with DIMS +3.50 D segments [46]. Binocular vision parameters remained largely stable, with no significant shifts in distance or near phoria, though a notable recession of the near point of convergence (NPC) by approximately 2 cm was observed. Positive fusional vergence at distance showed significant improvement in both break and recovery values after 12 months and continued to improve at 24 months. Stereopsis improved significantly at 12 months and was maintained throughout the study period. In terms of accommodation, a moderate reduction in accommodative lag was found alongside significant decreases in both monocular and binocular amplitude of accommodation, though these reductions remained within clinical norms. Negative relative accommodation decreased significantly, while positive relative accommodation was unchanged. Importantly, distance and near visual acuities improved significantly by 12 months and remained stable thereafter, indicating preserved or enhanced visual performance. Overall, the findings highlight that DIMS lenses not only effectively mitigate myopia progression but also maintain good binocular and accommodative function over two years, though continued monitoring of NPC is advised [46]. A recent study evaluated dynamic accommodation responses in both adults and children wearing three types of commercially available myopia control spectacle lenses (MiYOSMART, Stellest, and MyoCare), comparing centered and decentered viewing positions to assess the impact of peripheral defocus optics [47]. Across all tested conditions, including different lens designs and positions, there were no significant differences in accommodative lag or amplitude. Both adult and pediatric participants consistently exhibited minimal accommodative lag (<0.5 D) during near tasks, with stable and comparable response amplitudes irrespective of lens type or peripheral optics engagement. Further, experiments using regular single-vision lenses with central apertures designed to isolate peripheral refraction (introducing various defocus and astigmatic conditions) also showed no significant impact on dynamic accommodation patterns. Overall, the findings indicate that peripheral defocus modifications in these myopia control spectacles do not affect short-term accommodative behavior, suggesting that their myopia control effect likely operates through mechanisms independent of accommodation (Table 2).

## 4. Clinical Efficacy of Myopia Control Spectacle Lenses

Over the 6-year follow-up [11], children who continuously wore DIMS spectacle lenses (Group 1) demonstrated sustained myopia control, with a cumulative mean progression of −0.92 ± 1.15 D and axial elongation of 0.60 ± 0.49 mm, indicating an average annual progression rate markedly lower than typically reported in untreated children. Importantly, no significant difference in myopia progression was observed between the first three years and the subsequent three years, suggesting a stable, ongoing treatment effect. Children who switched from DIMSs to single-vision (SV) lenses after 3.5 years (Group 2) and those who reverted to SV lenses after wearing DIMS for 1.5 years (Group 4) showed increased myopia progression and axial growth compared to continuous DIMS users, yet no evidence of a rebound effect was found following discontinuation. Group 3, who started DIMSs after two years of SV lens wear, also exhibited robust myopia control, with even slower axial elongation in the final 2.5 years compared to Group 1 [11]. This represented roughly a 55–60% reduction in myopia progression rate with the DIMSs.

Essilor’s HALT lens (Stellest) was evaluated in a large two-year RCT in China. In this trial [12], 167 children (ages 8–13) were assigned to wear either highly aspherical lenslet (HAL) spectacles, slightly aspherical lenslet (SAL) spectacles, or single-vision lenses. The two-year outcomes, showed that the HAL (Stellest) lenses slowed myopia progression by ~0.80 D and axial growth by 0.35 mm versus single-vision controls [12]. This corresponds to about a 55% reduction in dioptric progression and 50% reduction in eye elongation, very comparable to DIMS efficacy. The SAL lenses also showed a significant but smaller treatment effect, confirming a dose–response: lenses with less asphericity (hence delivering a weaker defocus signal) produced less myopia control effect. One crucial finding was the impact of compliance—children who wore the Stellest (HALT) lenses full-time (at least 12 h per day) achieved an even greater slowing, up to 67% in refractive progression (+0.99 D less myopia) and 60% in axial elongation (+0.41 mm difference) over two years. This underscores that treatment dose matters: consistent wear maximizes efficacy, whereas part-time wear likely dilutes the effect. After the initial 2-year study, all participants were switched to HALT lenses for a third year, and a fresh control group was recruited for ethical comparison. The extended results showed that myopia control benefits persisted in year 3 for the original HALT group, and even the previously untreated children (who started HALT in year 3 at an older age) experienced significant slowing compared to the new controls. This suggests older children (up to mid-teens) can still benefit from starting spectacle treatment, echoing the DIMS crossover findings. Moreover, the 5-year randomized follow-up study evaluated the long-term efficacy of spectacle lenses with highly aspherical lenslets (HALT) for myopia control in children aged 8–13 years [11]. Compared to an extrapolated single-vision lenses (ESVL) control group, HALT significantly slowed spherical equivalent refraction (SER) progression by 58% (−1.27 D vs. −3.03 D; *p* < 0.001) and axial length (AL) elongation by 52% (0.67 mm vs. 1.40 mm; *p* < 0.001). The treatment effect was equivalent to preventing ~3 years of myopia progression. Long-term HALT use also reduced the incidence of high myopia (≥−6.00 D) from 38% in the ESVL group to 9% (*p* = 0.002). The ESVL group’s validity was confirmed by alignment of projected 3rd-year data with an actual single-vision control cohort (*p* > 0.83). Faster progression occurred during years 2–3, potentially linked to COVID-19 lockdowns. Younger baseline age predicted greater progression. These results demonstrate sustained efficacy of HALT spectacles in controlling myopia over 5 years and support extrapolated controls for long-term myopia studies [11].

The SightGlass DOT lenses have also been tested in a large-scale clinical trial known as CYPRESS. In a three-year double-masked RCT across multiple North American sites (*n* = 256 children, ages 6–10), the DOT 0.2 design (milder diffuser density) showed significant efficacy compared to single-vision glasses [32]. After 3 years, children wearing DOT 0.2 lenses had on average 0.33 D less myopic progression and 0.13 mm less axial elongation than controls [32]. Although the percentage reduction (~30%) was more modest than that seen with lenslet designs, the result was statistically significant. Interestingly, the higher scatter DOT 0.4 design did not show significant benefit in that trial, indicating that too much induced blur might have reduced wear time or had diminishing returns (those participants were switched to DOT 0.2 after 2 years). The study coincided with the COVID-19 pandemic, during which home confinement likely increased near work and reduced outdoor time; an analysis suggested the treatment effect was attenuated during lockdown periods for all groups [32]. An extension to a fourth year (with some control children continuing and the remaining switched to DOT) demonstrated an additional treatment benefit in year 4 for those who stayed on DOT 0.2, versus those who had only started it later. This reinforces that earlier and longer treatment is beneficial. At 4 years, no adverse events were noted [32].

For the ZEISS MyoCare (CARE) lenses, a randomized trial in China recently reported one-year results. Children 8–12 years old wearing ZEISS MyoCare (CARE) lenses had an average refractive progression of −0.56 D in a year, compared to −0.71 D with regular lenses, a difference of 0.15 D that did not reach statistical significance in year 1 [14]. However, axial elongation was 0.27 mm with CARE vs. 0.35 mm with SV, a difference of ~0.08–0.09 mm that was statistically significant. This corresponds to roughly a 25% reduction in axial growth over one year, although slightly lower in magnitude than DIMS/HALT over the same interval. The authors noted that a longer follow-up might show a larger refractive difference, as the eye’s refractive changes often lag behind axial length changes in short-term studies. No adverse events were reported, and visual acuity with CARE lenses was essentially the same as with single-vision lenses [14]. Ongoing studies (including a second-year outcome) will shed more light on the long-term efficacy of CARE lenses.

Taken together, the clinical data show that these advanced spectacle lenses slow myopia progression by approximately 30–60% on average, depending on the design and study population [11,14,32]. In practical terms, this means a child who might normally progress by say −1.00 D per year could instead progress by only −0.4 to −0.7 D per year with treatment. Over several years, the avoided myopia can add up to a diopter or more, potentially keeping a child from reaching high myopia [48]. It is noteworthy that the efficacy of the best spectacle treatments (DIMS, HALT) now approaches that of low-dose atropine [49], and of overnight ortho-k [50], or dual-focus [5] contact lenses. However, these approaches differ in mechanism, safety profile, and patient suitability. Table 3 summarizes the principal advantages and limitations of these modalities relative to spectacle-based strategies.

Although next-generation spectacle lenses have demonstrated consistent efficacy, several limitations must be acknowledged. Most large-scale randomized clinical trials were conducted in East Asian populations, where myopia incidence is exceptionally high, and may not fully reflect outcomes in other ethnic or environmental contexts. Study durations vary considerably (typically 1–3 years), and industry sponsorship may introduce potential bias in study design or reporting. Few independent, non-industry-funded replications are available, and direct head-to-head comparisons between lens types remain limited. Moreover, long-term (>10 year) outcomes and real-world effectiveness data are scarce. Future research should prioritize multicenter, ethnically diverse, and independently funded studies to confirm durability and generalizability of treatment effects.

## 5. Visual Performance, Compliance, and Safety Considerations

A critical aspect of any myopia control intervention is how it affects the wearer’s visual experience and quality of life, since poor tolerance could lead to non-compliance and reduced effectiveness [51]. Across the various spectacle lens trials, children’s visual performance with the new myopia control spectacle lenses has been remarkably good [11,12,52]. Distance and near visual acuity remain essentially normal: studies found no significant differences in high-contrast acuity between kids wearing DIMS or HALT lenses and those wearing regular lenses [11,12,52]. Low-contrast acuity and contrast sensitivity tests likewise showed minimal to no degradation with the lenslet or DOT designs [32]. For example, a sub-study on Essilor Stellest wearers reported no impact on distance acuity, near reading acuity, or stereoacuity compared to controls [53]. In the 4-year DOT trial, visual function outcomes (high/low contrast vision, stereopsis, reading speed) were comparable between the DOT 0.2 group and the single-vision group [32], indicating that even with deliberately reduced contrast, the effect was subtle enough not to hamper functional vision [33].

Subjectively, adaptation to these lenses should generally be quick and uneventful. With DIMS, HALT, and CARE lens wear, the majority of children report clear vision [11,14,32]. The CARE 1-year study noted no complaints of discomfort or adaptation issues—all participants could tolerate the lenses well [14]. These lenses may initially produce faint ghost images under certain conditions (e.g., at night looking at point lights) due to the lenslets [19], but children can typically neuro-adapt [54] and may not find it troublesome. The cosmetic appearance of these lenses is essentially similar to a single vision lens, an important factor for child and parent acceptance [55]. The micro-lenslets or diffusers are practically invisible when the glasses are worn; one has to hold the lenses up to light at certain angles to notice the patterns. This is a major improvement over something like executive bifocals, which have an obvious line. As a result, there is usually no social stigma or self-consciousness for the child—the glasses look like any regular pair [56].

Compliance has been reported to be high in these trials, which is a testament to good comfort and vision [11,14,32]. In the Stellest 2-year study [12], for instance, over 90% of participants were able to wear their glasses full-time (12 or more hours a day). Children as young as 6–8 adapt easily and often prefer these glasses over alternatives like contact lenses or atropine drops (which can have side effects like light sensitivity) [57]. One key practical advantage of spectacle-based myopia control is that it is non-invasive and low maintenance—no need for inserting lenses into the eyes or instilling drops nightly. Parents often find it easier to ensure a child wears glasses than to supervise contact lens hygiene or remember nightly eye drops [58]. This convenience likely boosts adherence to these spectacle lenses for myopia control. In long-term follow-ups, most children continued wearing the myopia control spectacles without issue, and many opted to stay in the study or continue the treatment by choice [11].

From a safety perspective, spectacle lenses are inherently very safe—there are none of the ocular health risks associated with contact lens use [59] or pharmacologic side effects from atropine [60]. The primary safety concerns would be if the lenses caused any delay in detecting refractive changes or if they induced any binocular vision stress [46]. On the first point, studies show that myopia still progresses in some treated children, so clinicians can adjust the prescription of the clear zone as needed [11,14,32]. The six-year DIMS study reported no adverse effects on visual function—acuity, accommodation, and convergence remained within normal range for all participants [11]. On the second point, a 24-month prospective study assessed changes in binocular vision and accommodation in 23 Malay myopic children (aged 7–12 years) wearing DIMS spectacle lenses. Significant changes included a median recession of the near point of convergence by 2 cm (*p* = 0.001), reduced monocular amplitude of accommodation by 1.67 D (*p* = 0.002), increased distance positive fusional vergence (break: +8Δ, *p* = 0.026; recovery: +6Δ, *p* = 0.033), improved stereopsis (*p* < 0.001), reduced accommodative lag by 0.25 D (*p* = 0.002), and an elevated AC/A ratio (*p* < 0.001). Myopia progression was slowed (spherical equivalent refraction: −0.35 ± 0.38 D; axial length elongation: 0.20 ± 0.22 mm), and visual acuity improved (*p* < 0.001). Clinically significant changes in NPC and distance PFV suggest adaptations to long-term lens wear. Regular NPC monitoring should be included as part of the follow up care to monitor visual comfort, though the absence of a control group in this specific study limits direct comparative inferences about DIMS-specific effects.

While most studies have focused on children with mild-to-moderate myopia, limited evidence is available for specific pediatric subgroups such as high myopes, anisometropes, or children with concurrent amblyopia or strabismus. Early reports suggest these lenses can be prescribed cautiously, with attention to binocular balance and adaptation. However, further investigation is needed to establish efficacy and safety in these populations. Clinicians should evaluate these cases individually, considering factors such as anisometropia magnitude, ocular alignment, and accommodative ability.

## 6. Global Perspectives and Future Directions

The advent of effective myopia control spectacles is a significant milestone in managing what has become a global public health challenge. Most clinical trials so far have been conducted in East Asian populations (China, Hong Kong, Japan) where childhood myopia prevalence is extremely high, often >80% by teenage years [61]. The robust efficacy demonstrated in these trials has led to rapid adoption of products like MiYOSMART [62] and Stellest [25] in dozens of countries worldwide, including across Asia, Europe, and the Americas, and have been integrated into myopia management programs. Notably, the DOT lens trials in the North America indicate that children of Caucasian descent also respond to the treatment (with slower progression vs. controls) [32]. This suggests the efficacy is not ethnicity-specific but rather tied to fundamental visual physiology. However, it will be valuable to gather more data in diverse populations—for instance, in Europe and Australia with different environmental contexts and healthcare systems. There may be regional differences in myopia progression rates (due to lifestyle, nearwork, or genetics) that could influence the absolute effect sizes observed [63,64].

One interesting aspect is the age factor [65]. Most trials enrolled kids around 8–13 at start, aligning with typical onset of school myopia [11,12]. The extensions in DIMS and HALT studies showed that even as children get older (14–16 years), those who continue treatment still progress slower than those without treatment [11,12]. This addresses a prior concern that maybe the treatment would only “buy time” and that once the child hits teenage years, the effect might diminish or the eye might “catch up” in growth—which does not appear to be the case, at least up to late teens [48]. More data is needed on children who start treatment very young (<6 years) or on those who might benefit in late teenage years. Future research might clarify if there is any merit in continued wear into early adulthood for those still progressing.

A recent network meta-analysis suggests all these new designs cluster around ~50% efficacy [66]. A direct head-to-head trial (for example, DIMSs vs. HALT in the same population) would be valuable. Another frontier is combining spectacle lenses with other myopia control treatments. Early studies combining DIMS glasses with low-dose atropine (0.01% or 0.05%) have shown additive effects [67,68]. In a prospective non-randomized study of 146 European children (aged 6–18 years), DIMS spectacles, 0.01% atropine, and their combination were compared over 12 months [67]. All interventions reduced axial elongation and SER progression versus SV controls (*p* < 0.001). The combination group showed the greatest efficacy: 77% reduction in axial elongation and 70% in SER progression versus controls. Atropine and DIMS monotherapy each reduced progression by ~57–62%. Notably, 18% of combination-treated participants had no axial elongation. The study highlights the additive effect of combining optical and pharmacological interventions in a European cohort, though non-randomized allocation limits generalizability. More recent studies combining DIMS or HALT lenses with low-dose atropine (0.0025%) [66] have shown additive effects in slowing myopia progression, and several multicenter clinical trials are underway to evaluate optimal concentrations, long-term safety, and potential rebound effects. Although these optical–pharmacologic combinations are still considered off-label in most jurisdictions, regulatory agencies in Asia, Europe, and North America are actively reviewing emerging data.

The global adoption of myopia control spectacle lenses depends not only on their demonstrated clinical efficacy but also on practical considerations such as accessibility, affordability, and long-term monitoring. These technologies are currently distributed primarily through specialty optical channels, with costs typically ranging between USD 250 and 450 per pair depending on region, and limited insurance reimbursement remains a barrier in many markets. Broader integration into public health systems, local manufacturing partnerships, and practitioner training could help reduce cost barriers and improve access, particularly in low- and middle-income countries. Finally, while current longitudinal studies document sustained efficacy for up to six years, the lack of decade-long data underscores the need for extended prospective surveillance to confirm long-term safety, durability of treatment effect, and refractive stability into adulthood.

## 7. Conclusions

Spectacle lenses for myopia control have ushered in a new era where myopia progression can be safely and significantly slowed with a simple pair of glasses. The optical principles of myopic defocus [67] and contrast modulation [31] have been utilized by these next-generation spectacle lenses, the efficacy of which has been reported by multiple clinical trials showing reduced axial elongation in children. With outcomes on par with other modalities [57], these lenses represent a promising and evidence-based approach that may become an important component of myopia control practice worldwide. Ongoing research and development, along with long-term monitoring of current wearers into adulthood, will continue to refine our understanding.

## Figures and Tables

**Table 1:** Key Design Features and Efficacy of Leading Myopia Control Spectacle Lenses.

Lens Design (Brand)	Optical Features	Proposed Mechanism	Study Population/Follow-Up	Clinical Efficacy vs. Single Vision
DIMS—Defocus Incorporated Multiple Segments (MiyoSmart^®^, Hoya Lens, Tokyo, Japan)	~400 lenslets (+3.50 D each) in a honeycomb array; 9 mm central clear zone.	Simultaneous myopic defocus across peripheral retina (dual-focus optics).	*n* = 160; 2-year RCT; Follow-up 6 years.	0.44 D less myopia progression and 0.34 mm less axial elongation vs. control (≈60% reduction). Efficacy sustained through 3–6 years with no rebound.
HALT—Highly Aspherical Lenslet Target (Stellest^®^, Essilor, Charenton-le-Pont, France))	1021 contiguous aspherical lenslets arranged in 11 concentric rings; 9 mm central clear zone. Each ring’s lenslets have differing aspheric power profiles.	“Volume of myopic defocus” created by aspheric lenslets (simultaneous multi-depth defocus signal).	*n* = 167; 2-year RCT; Follow-up 5 years.	0.80 D less progression and 0.35 mm less elongation vs. control (≈55% reduction). Higher wear compliance (≥12 h/day) increased efficacy to 67% (0.99 D, 0.41 mm). Sustained effect ~50% over 5–6 years (no loss of treatment effect).
DOT—Diffusion Optics Technology (SightGlass Vision, Dallas, TX, USA)	Thousands of microscopic diffusive “dots” (~0.1–0.2 mm); 5 mm central clear zone. Two refractive indices in lens to scatter light and reduce contrast.	Mild uniform reduction in retinal image contrast (especially high-frequency details), mimicking outdoor visual conditions.	*n* = 256; 3-year RCT; Follow-up 4 years.	Slowed axial growth by 0.13 mm and refractive progression by 0.33 D vs. control (≈30% less progression). Demonstrated continued benefit at 4 years (additional slowing vs. control). No significant differences in acuity or comfort compared to SV lenses.
CARE—Cylindrical Annular Refractive Element (MyoCare^®^, Zeiss Vision Care, Aalen, Germany)	Multiple ring-shaped micro-optic zones alternating with normal vision zones (approximately 50/50 area split); central distance zone (~8–10 mm).	Concentric annular defocus zones impose myopic defocus, while clear zones maintain central vision (balanced defocus and clarity).	*n* = 150; 1-year RCT; Follow-up 2 years (ongoing).	Axial elongation 0.27 mm (CARE) vs. 0.35 mm (SV)—a 0.08–0.09 mm reduction (~25%). Refractive progression 0.14 D less than control (not statistically significant at 1 year). Well tolerated with no adaptation issues.

**Table 2:** Optical and Biological Mechanisms Proposed for Myopia Control Spectacle Lenses.

Mechanism	Description	Supporting Evidence	Representative Lens Designs
Myopic defocus	Part of the image is intentionally focused slightly in front of the retina to signal the eye to slow its axial growth.	Strong evidence from animal studies and multiple randomized controlled trials showing reduced myopia progression.	Defocus Incorporated Multiple Segments (DIMS), Highly Aspherical Lenslet Target (HALT), Cylindrical Annular Refractive Element (CARE) lenses
Contrast modulation	Light scattering or diffusion is used to gently reduce image contrast across the retina, mimicking outdoor visual conditions that are protective against myopia.	Confirmed by clinical trials demonstrating effective myopia control without optical power addition.	Diffusion Optics Technology (DOT) lenses
Induced optical aberrations	Small, controlled distortions in peripheral image quality alter retinal signaling and may contribute to slower eye growth.	Supported by laboratory and modeling studies; effect is secondary to defocus.	DIMS and HALT lenses
Accommodation and binocular stability	Lens designs maintain normal focusing and eye-alignment responses despite peripheral optical modifications.	Clinical studies show no adverse impact on focusing or binocular coordination.	DIMS, HALT, and CARE lenses

**Table 3:** Comparative Overview of Major Myopia Control Interventions.

Intervention	Mean Reduction in Axial Elongation	Efficacy Summary	Safety Profile	Adherence/Practical Considerations
DIMS/HALT/CARE Spectacle Lenses	40–60%	Comparable efficacy to atropine and orthokeratology in multiple RCTs.	Excellent safety; no ocular adverse events reported.	Very high adherence; easy adaptation, worn like regular spectacles.
Low-Dose Atropine (0.01–0.05%)	50–60%	Dose-dependent response; higher concentrations show greater efficacy.	Mild photophobia and near blur possible; generally well tolerated.	Very good adherence; nightly instillation required; potential rebound after discontinuation.
Orthokeratology	50–60%	Robust short-term efficacy on axial growth; long-term data consistent.	Small but measurable risk of microbial keratitis; requires hygiene compliance.	Moderate adherence; overnight lens wear; requires regular monitoring.

## Data Availability

Not applicable.

## Abbreviations

The following abbreviations are used in this manuscript:

CARE	Cylindrical Annular Refractive Element
DIMS	Defocus Incorporated Multiple Segments
DOT	Diffusion Optics Technology
HALT	Highly Aspherical Lenslet Target
PAL	Progressive Addition Lens
SV	Single Vision
SER	Spherical Equivalent Refraction
HOAs	Higher-Order Aberrations
NPC	Near Point of Convergence
RCT	Randomized Controlled Trial
AL	Axial Length
SE	Spherical Equivalent
